# The effect of imaging modality (magnetic resonance imaging vs. computed tomography) and patient position (supine vs. prone) on target and organ at risk doses in partial breast irradiation

**DOI:** 10.1002/jmrs.453

**Published:** 2020-12-07

**Authors:** Emily Brown, Kylie Dundas, Yolanda Surjan, Daniela Miller, Karen Lim, Miriam Boxer, Verity Ahern, George Papadatos, Vikneswary Batumalai, Jennifer Harvey, Debra Lee, Geoff P. Delaney, Lois Holloway

**Affiliations:** ^1^ Medical Radiation Science (MRS) School of Health Sciences The University of Newcastle Callaghan NSW Australia; ^2^ Liverpool and Macarthur Cancer Therapy Centers Liverpool NSW Australia; ^3^ Ingham Institute for Applied Medical Research Liverpool NSW Australia; ^4^ South Western Sydney Clinical School University of New South Wales Sydney NSW Australia; ^5^ Crown Princess Mary Cancer Centre Westmead Hospital Sydney NSW Australia; ^6^ Westmead Clinical School University of Sydney Sydney NSW Australia; ^7^ School of Medicine University of Queensland Brisbane QLD Australia; ^8^ Princess Alexandra Hospital Brisbane QLD Australia; ^9^ School of Medicine University of Western Sydney Sydney NSW Australia; ^10^ Centre for Medical Radiation Physics Faculty of Engineering and Information Sciences University of Wollongong Wollongong NSW Australia

**Keywords:** Magnetic resonance imaging, organs at risk, partial breast irradiation, patient positioning, X‐ray computed tomography

## Abstract

**Introduction:**

Conventionally computed tomography (CT) has been used to delineate target volumes in radiotherapy; however, magnetic resonance imaging (MRI) is being continually integrated into clinical practice; therefore, the investigation into targets derived from MRI is warranted. The purpose of this study was to evaluate the impact of imaging modality (MRI vs. CT) and patient positioning (supine vs. prone) on planning target volumes (PTVs) and organs at risk (OARs) for partial breast irradiation (PBI).

**Methods:**

A retrospective data set, of 35 patients, was accessed where each patient had undergone MRI and CT imaging for tangential whole breast radiotherapy in both the supine and prone position. PTVs were defined from seroma cavity (SC) volumes delineated on each respective image, resulting in 4 PTVs per patient. PBI plans were generated with 6MV external beam radiotherapy (EBRT) using the TROG 06.02 protocol guidelines. A prescription of 38.5Gy in 10 fractions was used for all cases. The impact analysis of imaging modality and patient positioning included dose to PTVs, and OARs based on agreed criteria. Statistical analysis was conducted though Mann–Whitey U, Fisher’s exact and chi‐squared testing (*P* < 0.005).

**Results:**

Twenty‐four patients were eligible for imaging analysis. However, positioning analysis could only be investigated on 19 of these data sets. No statistically significant difference was found in OAR doses based on imaging modality. Supine patient position resulted in lower contralateral breast dose (0.10Gy ± 0.35 vs. 0.33Gy ± 0.78, p = 0.011). Prone positioning resulted in a lower dose to ipsilateral lung volumes (10.85Gy ± 11.37 vs. 3.41Gy ± 3.93, *P* = <0.001).

**Conclusions:**

PBI plans with PTVs derived from MRI exhibited no clinically significant differences when compared to plans created from CT in relation to plan compliance and OAR dose. Patient position requires careful consideration regardless of imaging modality chosen. Although there was no proven superiority of MRI derived target volumes, it indicates that MRI could be considered for PBI target delineation.

## Graphical abstract

This study investigates how image modality (magnetic resonance imaging (MRI) vs computed tomography (CT)) and patient positioning (supine vs. prone) affects partial breast irradiation (PBI) planning. There was no proven superiority of MRI‐derived target volumes, although notably, positioning influenced dose to organs at risk (OARs). MRI has the potential to be integrated into future practice to replace the need for CT based planning.

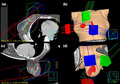

## Introduction

The rationale for adjuvant radiotherapy is to destroy potential microscopic disease foci in the residual breast after breast conserving surgery (BCS).^1^ Increasing cancer survivorship means more women are living longer after initial diagnosis and are at risk of long‐term side effects of radiation treatment.^1^, ^2^ These may include cardiac disease,^3^ pulmonary fibrosis,^4^ rib fractures^2^ and/or the potential for radiation induced secondary malignancies.^2^

There has been increasing interest in delivering radiation to the site of the tumour bed alone, known as partial breast irradiation (PBI) for patients with ‘low‐risk’, lymph node‐negative breast cancer.^5^, ^6^ This technique delivers therapeutic dose to the seroma with a margin, as opposed to irradiation of the entire ipsilateral breast, as seen in whole breast irradiation (WBI).^7^, ^8^ A smaller treatment volume allows hypofractionation of external beam radiation therapy (EBRT) and aims to reduce dose to organs at risk (OARs), such as the contralateral breast, lungs and heart.^9^ Clinical trial evidence^10^, ^11^, ^12^ has demonstrated non‐inferiority, in terms of local relapse and equivalent or fewer late normal tissue complications. This applied to a select subset of patients (age > 50, 0‐3 involved axillary nodes, grade 1‐3 disease and with tumours ≤ 3 cm). This is supported by similar patient selection criteria in additional reviews and long‐term trial updates on PBI delivery.^13^, ^14^ The use of PBI is demonstrated further by additional trials^13^, ^14^ and the development of protocol and consensus guidelines within the United States^5^ and the United Kingdom.^6^

Target volumes have been typically derived from computed tomography (CT). The use of magnetic resonance imaging (MRI) for defining radiotherapy treatment volumes has gained increased interest for breast cancer due to superior resolution between glandular breast and adipose tissue^15^ as the result of intrinsic soft tissue visualisation properties.^16^, ^17^ Supine position is most common for breast radiotherapy;^7^ however, most breast MRI is performed with the patient in the prone position, to achieve the best signal to noise ratio.^18^ Reported dosimetric benefits of the prone position in breast radiotherapy include improved dose distribution in women with large pendulous breasts,^19^ due to increased distance between targets and OARs, resulting in reduction of dose to healthy tissues such as the ipsilateral lung.^19^, ^20^ Use of this positioning method varies across clinical centres and countries. ^7^, ^21^ The feasibility of MRI integration into breast cancer radiotherapy planning and treatment has been demonstrated through recent investigations (with the patient in both the supine and prone position).^22^, ^23^ Furthermore, seroma cavities are smaller when derived from MRI, compared to CT due to clearer seroma definition.^24^ For these reasons, integrating MRI into the radiotherapy planning pathway may result in a smaller region of the breast receiving radiation.

At the time of project commencement, to our knowledge, there was no published data that had evaluated the dosimetric implications of chosen image modality (MRI vs. CT) and patient positioning on target and OAR doses in PBI planning. Therefore, the primary aim investigated if PTV and OAR doses differed when PBI planning is based on target volumes derived from MRI when compared to CT. A secondary aim investigated if patient positioning (supine vs. prone) had an effect on the OAR differences due to imaging modality chosen for target delineation in PBI.

## Methods and Materials

Following ethics approval (South Western Sydney Local Health District Human Research Ethics Committee (HREC number: HREC/16/LPOOL/603)), a retrospective cohort of patient data collected from previous investigations, was accessed.^24^

### Context

**Figure 1:**
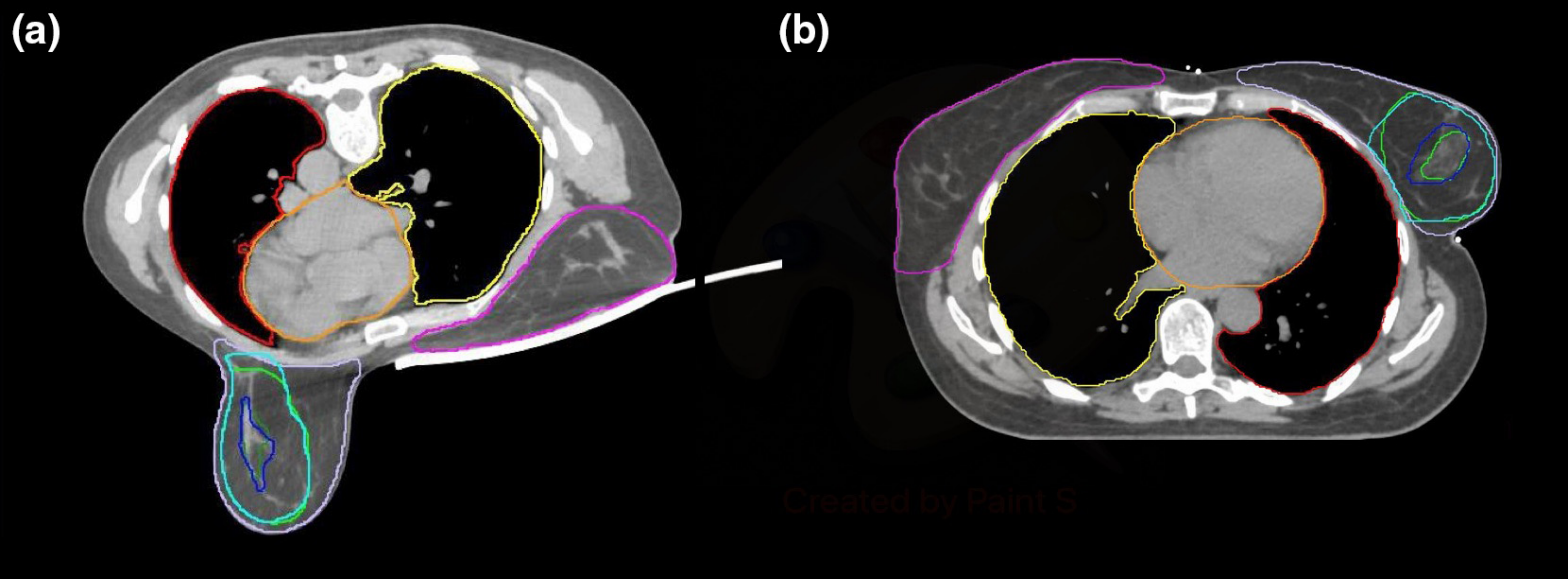
Typical CT image sets and contour information available for this investigation. A: Prone, transverse view B: corresponding transverse view, Red contour: Ipsilateral lung, Yellow contour Contralateral Lung, Orange contour: Heart, Pink contour: Contralateral Breast, Lilac contour: Ipsilateral Breast, Dark Blue contour: Seroma Cavity defined on MRI, Dark Green contour: Seroma Cavity defined on CT, Light Blue contour: planning target volume used for evaluation based off MRI seroma cavity, Light Green contour: planning target volume used for evaluation based off CT seroma cavity.

The cohort of 35 patients received adjuvant radiotherapy after BCS between July 2012 and November 2014. Each patient underwent MRI and CT scans in supine and prone positions, resulting in 4 image data sets per patient. Supine positioning included the utilisation of a vac bag with flat wingboard and arms over head. Prone position used a 16‐channel Sentinelle Breast MRI System for both CT and MRI scans. Further details are provided in previous work, including patient exclusion based on body habitus.^24^ CT scans were performed on Phillips CT scanner (Phillips Healthcare, The Netherlands), and MRI was conducted on a MAGNETOM Skyra 3T (Siemens Medical Systems, Erlangen, Germany). Patients were positioned under a coil bridge with a surface coil for the supine MRI scans and using the Breast MRI System for the prone MRI Scans.^22^, ^24^ Scans were conducted without breathing restrictions. Each patient’s 4 image sets had a final seroma cavity (SC) volume that was the result of a Simultaneous Truth And Performance Level Estimation (STAPLE) algorithm^25^, ^26^ of 11 different SC contours (delineated by nine radiation oncologists and two radiologists). Subsequently, supine and prone MRI data sets were rigidly registered to the corresponding CT, to allow radiation therapy planning. The determination of these volumes and the image registration technique has been described in previous work.^24^, ^27^ The supine and prone CT image sets each containing CT and MRI determined SC volumes and OAR volumes as determined from CT were used for this study. Figure 1 depicts a typical CT image set and contour information that was available for this study.

### Patient selection

From the initial cohort of 35 patients described above, the following criteria were used to determine eligibility for this study; age ≥ 50 years at time of diagnosis and pathology ≤ 3.0 cm (length as stated on histology report). This was based on international consensus statements^5^, ^6^ and published clinical trial data^10^, ^11^, ^12^. Laterality, topography (breast quadrant as per ICD‐03 topography site notes),^28^ age at diagnosis and size of breast were recorded in the patient notes. Other factors typically considered for PBI EBRT eligibility such as histology were not considered as this was primarily a planning investigation, and these characteristics should not affect geometry or dosimetry.

### Radiotherapy treatment planning

TROG 06.02 guidelines^29^ were followed for the radiotherapy treatment planning aspects of this study. For each data set, clinical target volumes (CTV) and planning target volumes (PTV) were generated from SC volumes. Target expansions and OAR structures used in this investigation are outlined in Table 1.

**Table 1:** Definition and Dose compliance for targets and OARs

Target/ OARs	Definition	Metric
PB CTV	SC (excluding 5mm inside the patient contour and clipped at the interface of the breast tissue and pectoralis major muscle) + 10mm	
PB PTV	PB CTV + 10mm	
PTV EVAL	PB PTV excluding lungs, heart, ribs and pectoralis muscle. Contour clipped 5mm inside the patient contour and was used for DVH assessment.	At least 90% of the prescribed dose (D_90_) should cover 95% of the target volume (PTV EVAL V_95%_) and the maximum dose delivered to 2 cm^3^ should not exceed 105% of the prescribed dose (D_105_) At least 90% of the prescribed dose (D_90_) should cover 90‐94% of the target volume (PTV EVAL V_90_) and the maximum dose delivered to 2 cm^3^ should not exceed 106‐110% of the prescribed dose (D_110_) (Minor Violation)
IpsiLung†	Ipsilateral lung as seen on CT. Created in previous investigations	Less than 10% of the volume (IpsiLung V_10_) receives less than 30% of the prescribed dose (D_30_)
ContraLung†	Contralateral lung as seen on CT. Created in previous investigations	Less than 10% of the volume (Contralateral Lung V_10_) receives less than 5% of the prescribed dose (D_5_)
Heart†	Heart as seen on CT. Created in previous investigations	Less than 5% of the volume (Heart V_5_) receives less than 5% of the prescribed dose (D_5_) (regardless of laterality)
IpsiBreast	Included STAPLE whole breast contour delineated in previous work image modality dependent	
IpsiBreastPRV†	Ipsilateral Breast excluding PB PTV	Less than 35% (IpsiBreast PRV V_35_) of the volume is covered by 95% of the prescribed dose (D_95_) and less than 60% of the volume is covered by 50% of the prescribed dose (D_50_)
ContraBreast†	Contralateral Breast as seen on CT. Created in previous investigations	Less than 5% of the prescribed dose (D_5_) is delivered to any point of the contralateral breast
Normal Tissue	Whole patient contour excluding PB PTV.	

PB CTV: partial breast clinical target volume, SC: Seroma Cavity, PTV EVAL: planning target volume used for evaluation, DVH: dose volume histogram, IpsiLung: ipsilateral lung, ContraLung: contralateral lung, IpsiBreast: ipsilateral breast, IpsiBreastPRV: ipsilateral breast planning reference volume contour excluding the PTV structure, ContraBreast: contralateral breast. ^†^OARs did not have minor violation criteria. Any plan with OAR dose exceeding described metrics was classed as non‐compliant.

**Figure 2:**
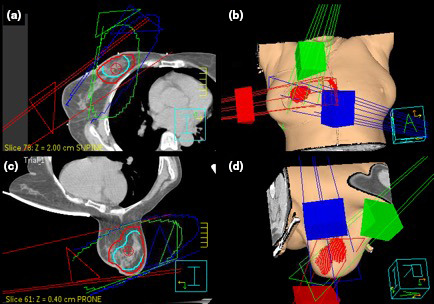
Comparison of planning techniques in supine and prone position. A: Supine, Transverse view of beam arrangement and target structure, B: Supine, 3D render of beam arrangement and target structure, C: Prone, Transverse view of beam arrangement and target structure, D: Prone, 3D render of beam arrangement and target structure, Red contour: planning target volume used for evaluation, Light Blue contour: clinical target volume.

This planning study utilised a 3 field non‐coplanar beam arrangement, as recommended by the TROG 06.02 study protocol.^30^ The direction of beam entry varied with patient position. In the supine position, this technique consisted of one lateral tangential beam, one superior medial beam and one inferior medial beam. The lateral beam was placed as close to horizontal as possible without exiting through the contralateral breast. The couch position was angled on the two medial beams to deliver dose from the superior and inferior directions, respectively. In the prone position, the beam arrangement consisted of two lateral beams and one medial beam. A limited range of gantry angles were available for the non‐coplanar beams to avoid potential gantry and couch collision. An example of the beam arrangement for a patient with right‐sided SC, in the supine and prone position, is demonstrated in Fig. 2.

Plans were generated using 6MV EBRT with wedges for dose optimisation. A prescription of 38.5Gy in 10 fractions was used for all cases.^29^, ^30^ All planning calculations were performed on CT data set utilising a 0.25 dose grid and the collapsed cone convolution (CCC) calculation algorithm within Pinnacle (version 9.10, Phillips, Netherlands).^31^ Manual optimisations of each plan were performed with the intent to meet the TROG 06.02 trial compliance criteria and are outlined in Table 1. Plans were categorised as compliant when all dose constraints in Table 1 were met. The category of minor violation was only applicable for planning target volume evaluation (PTV EVAL) structures. A plan was categorised as non‐compliant if at least one OAR objective was not met. Random plans, that is not all plans, were reviewed for quality assurance (QA), where one of two experienced radiation therapists randomly chose and selected plans to assess based on quality, clinical deliverability and protocol compliance. Additional QA checks were conducted for difficult/non‐compliant cases.

### Data collection and analysis

Plan compliance or non‐compliance was based on criteria outlined in Table 1 using scorecard features in Pinnacle. Additionally, Pinnacle was also used to record the volume of the contoured ipsilateral breast volume and PTV. These values were used to generate the PBI volume ratio, which was a ratio between ipsilateral breast volume to PTV EVAL volume. Digital imaging and communication in medicine (DICOM) files containing; structure, plan and dose data of completed plans were exported to MIM Maestro (MIM Software Inc., OH, USA) (MIM). Plans were assessed on further dose metrics outlined in Table 2, this assessment criteria was based on dose metrics outlined in previous investigations to ensure robustness of plan design.^10^, ^11^, ^12^, ^30^ Dose metric data were exported to Microsoft Excel (Microsoft Excel 2019, Microsoft Corporation, USA) for review. Organised data were then analysed using IBM SPSS Statistics Version 26.

**Table 2:** Dose metrics exported from MIM

Target/ OAR	Metric	Definition
CTV	CTV D_95%_	The dose received to 95% of the CTV, as a percentage of prescribed dose
PTV EVAL	PTV D_90%_	Dose received to 90% of the PTV EVAL, as a percentage of prescribed dose
		Dose delivered to 2 cm^3^
	PTV D_120%_	Any volume receiving 120% of dose
Ipsilateral Lung	IpsiLung D_30%_	Dose received to 30% of the Ipsilateral Lung, as a percentage of prescribed dose
Ipsilateral Breast	IpsiBreastPRV D_50%_	Dose received to 50% of the Ipsilateral Breast, as a percentage of prescribed dose
	IpsiBreastPRV D_95%_	Dose received to 95% of the Ipsilateral Breast, as a percentage of prescribed dose
	IpsiBreastPRV D_100%_	Dose received to 100% of the Ipsilateral Breast, as a percentage of prescribed dose
Heart	Heart D_5%_	Dose received to 5% of the Heart, as a percentage of prescribed dose
Contralateral Lung	ContraLung D_5%_	Dose received to 5% of the Contralateral Lung, as a percentage of prescribed dose
Contralateral Breast	ContraBreast D_5%_	Dose received to 5% of the Contralateral Breast, as a percentage of prescribed dose
	ContraBreast D_3%_	Dose received to 3% of the structure, as a percentage of prescribed dose
Normal Tissue	Patient‐PTV	Maximum dose delivered to normal structures outside PTV

OAR: organ at risk, CTV: clinical target volume, PTV EVAL: planning target volume used for evaluation, IpsiLung: ipsilateral lung, IpsiBreastPRV: ipsilateral breast planning reference volume excluding the PTV structure, ContraBreast: contralateral breast, ContraLung: contralateral lung, Normal Tissue: patient contour excluding PTV.

To assess the impact of image modality (MRI vs. CT) for target delineation, non‐parametric testing was employed. Mann–Whitney U tests were completed to show if there was significant variations in doses received by OARs between groups (MRI vs. CT). This method of analysis was also used to analyse dose discrepancies in individual OARs between different positioning methods (supine vs. prone). To determine if other factors such as topography, laterality, breast volume or seroma size influenced plan compliance, categorical analysis was performed. Separate chi‐squared tests were performed between the MRI and CT group to test for a relationship in resulting plan compliance. This method of testing was also used to determine if an association existed between supine and compliant plan or prone and compliant plans. Chi‐squared tests were also used to test for an association between PBI breast volume ratio vs. plan compliance, seroma size vs. plan compliance and laterality vs. plan compliance. Fisher’s exact test and chi‐squared tests were performed to assess for a relationship between ranked ipsilateral breast volume (<500 ml, 501–1000 ml and >1000 ml) and plan compliance. To determine if topography influenced plan compliance, specifically volumes located in the upper inner quadrant (UIQ), a Fisher’s exact test was used. A *P* value of < 0.05 was used to denote significance for all tests.

## Results

After PBI patient selection criteria was applied to the initial cohort, 24 patients were deemed eligible for this study. Table 3 displays the patient characteristics, which includes seroma laterality, tumour position and age at diagnosis. Each patient had 4 SC volumes resulting in 96 possible plans. However, from the PBI eligible cohort, a further four patients prone data sets were omitted from the positioning arm due to inadequate alignment of the patient’s sternum on the prone breast board. This resulted in inconsistent and incorrect positioning of the contralateral breast and has been documented in previous publications^21^, ^27^. A further patient’s prone data sets were removed due to the absence of a prone STAPLE SC volumes. In total, there were 86 data sets (24 patients) included in the analysis of MRI vs. CT and 76 data sets (19 patients) included in the supine vs. prone comparison. Mann Whitney U testing of PTV EVAL did not display a statistical significance for size discrepancies between modalities (MRI 144 cc^3^ ± 86 and CT 173 cc^3^ ± 96 *P* = 0.051).

**Table 3:** Patient Characteristics

		Imaging Modality Analysis (*n* = 24 patients (86 data sets))	Positioning Analysis (*n* = 19 patients (76 data sets))
Laterality	Left	40	36
	Right	46	40
Topography†	UOQ	40	36
	UIQ	20	20
	LOQ	6	4
	LIQ	4	4
	Intraductal breast	4	0
	Central portion of breast	4	4
	Unspecified	8	8
Age	Mean (Years)	63	62
	Range (Years)	50‐72	50‐72
Breast size	<500ml	32	31
(IpsiBreast)	501‐1000ml	39	34
	>1000ml	15	11

UOQ: upper outer quadrant, UIQ: upper inner quadrant, LOQ: lower outer quadrant, LIQ: lower inner quadrant: † Breast quadrant recorded in patient notes as per ICD‐03 topography site codes^28^.

### Plan compliance

Plan compliance rates are displayed in Table 4. MRI supine plans resulted in the highest plan compliance rate, of 88%, CT supine had a plan compliance rate of 83%. CT prone had an 84% plan compliance rate and MRI prone had a plan compliance rate of 79%. However, in an analysis of all 86 plans there was no statistical association between image modality and plan compliance; both CT and MRI resulted in 36 compliant and 7 non‐compliant plans each. There was also an equal number of compliant plans when positioning was compared, with both positions resulting in 31 compliant plans and 7 non‐compliant each.

**Table 4:** Plan characteristics and resulting compliance

ID	Laterality	Topography	MRI						CT					
			Supine			Prone			Supine			Prone		
			Breast Volume (ml)	Ratio	Plan Compliance	Breast Volume (ml)	Ratio	Plan Compliance	Breast Volume (ml)	Ratio	Plan Compliance	Breast Volume (ml)	Ratio	Plan Compliance
PBI01	LEFT	UOQ	1262	0.26	✓	‐	‐	‐	1262	0.30	✓	‐	‐	‐
PBI02	RIGHT	UOQ	792	0.17	✓	‐	‐	‐	722	0.39	✓	‐	‐	‐
PBI04	RIGHT	LOU	1096	0.16	✓	1156	0.16	✓	1103	0.11	✓	1156	0.16	✓
PBI05	LEFT	UOQ	543	0.37	✓	595	0.35	✓	595	0.26	✓	649	0.25	✓
PBI07	LEFT	UIQ	489	0.18	X^‡§^	566	0.21	X^‡¶^	489	0.26	X^‡§¶^	573	0.29	X^‡§¶^
PBI08	RIGHT	UOQ	818	0.20	✓	926	0.16	✓	857	0.19	✓	898	0.21	✓
PBI09	LEFT	UIQ	460	0.19	X^‡§^	538	0.26	X^‡§^	482	0.19	X^‡§¶^	566	0.24	✓†
PBI10	RIGHT	UIQ	300	0.22	✓	270	0.52	✓	331	0.24	✓	305	0.39	✓†
PBI11	LEFT	Unspecified	698	0.16	✓†	951	0.16	✓†	744	0.33	X‡	932	0.43	X^‡•^
PBI12	LEFT	UOQ	733	0.20	✓	807	0.24	✓	737	0.21	✓	805	0.20	✓
PBI14	RIGHT	Unspecified	976	0.12	✓	1165	0.09	✓	1003	0.10	✓	1140	0.30	✓
PBI17	RIGHT	UIQ	890	0.25	✓	949	0.24	✓	911	0.22	✓	1000	0.21	✓
PBI19	LEFT	Intraductal	434	0.23	✓	‐	‐	‐	533	0.21	✓	‐	‐	‐
PBI20	RIGHT	UIQ	501	0.19	✓	632	0.22	✓	554	0.17	✓	561	0.29	✓
PBI22	RIGHT	UOQ	454	0.28	✓	458	0.30	✓	494	0.29	✓	566	0.28	✓
PBI23	RIGHT	UOQ	1102	0.10	✓	1402	0.10	✓	1129	0.17	✓	1415	0.10	✓
PBI25	LEFT	LIQ	298	0.16	X^‡§^	332	0.19	X^‡§^	324	0.18	X^‡§^	339	0.29	X^‡^
PBI26	RIGHT	LOQ	1703	0.32	✓	‐	‐	‐	1706	0.33	✓	‐	‐	‐
PBI27	RIGHT	Intraductal	750	0.16	✓	‐	‐	‐	819	0.17	✓	‐	‐	‐
PBI28	RIGHT	Central	376	0.16	✓	419	0.16	✓	400	0.37	✓	429	0.31	✓
PBI32	LEFT	UOQ	230	0.33	✓	265	0.18	✓	233	0.33	✓	259	0.24	✓
PBI33	LEFT	UOQ	422	0.50	✓	438	0.55	X^‡•^	486	0.43	✓	486	0.42	✓†
PBI34	RIGHT	UOQ	581	0.15	✓	603	0.19	✓	602	0.22	✓	608	0.21	✓
PBI35	LEFT	UOQ	398	0.26	✓	438	0.21	✓	418	0.30	✓	463	0.39	✓
			Plan Compliance Rate		88%	Plan Compliance Rate		79%	Plan Compliance Rate		83%	Plan Compliance Rate		84%

LIQ: lower inner quadrant, LOQ: lower outer quadrant, PTV: planning target volume, UIQ: upper inner quadrant, UOQ: upper outer quadrant, ✓: Compliant plan, X Non‐compliant plan, †: Minor Violation, ‡: Heart dose in violation, §: Ipsilateral Lung dose in violation, ¶: Contralateral Breast dose in violation, ^•^: IpsiBreast‐PTV in violation, Breast Volume: volume of ipsilateral breast contoured in pinnacle, Ratio: ratio of ipsilateral breast volume compared to PTV EVAL in pinnacle, Compliance: plan within constraints and assessed to meet proposed criteria, Topography: Breast quadrant recorded in patient notes as per ICD‐03 topography site codes^28^.

In all non‐compliant plans, the laterality of tumour was left sided and heart dose limitations were unable to be met. Other correlations for non‐compliance included observations of small ipsilateral breast volume and tumour located in UIQ. In a comparison of compliant and non‐compliant plans, ipsilateral breasts < 500 ml resulted in 72% compliance (23/32 plans), 501–1000 ml resulted in 87% compliance (34/39 plans) and > 1000 ml resulted in 100% compliance (15 plans). In an analysis of all data sets, left sided tumour volumes displayed an influence on compliance rates (14/14 non‐compliant plans) when compared with a right‐sided tumour volume (0/14 non‐compliant plans) (p = <0.001). Breast volume < 500 ml was shown to influence compliance rate (9/14 non‐compliant plans) when compared to breast volumes > 500ml (5/14 non‐compliant plans) (p = 0.022). A seroma located in the UIQ (7/14 non‐compliant plans) was observed to influence plan compliance when compared to other locations of breast topography including in the lower inner quadrant (LIQ) (4/14 non‐compliant plans), upper outer quadrant (UOQ) (1/14 non‐compliant plans) and unspecified parts of the breast (2/14 non‐compliant plans) (*P* = 0.016). Factors with no proven significance include seroma volume when categorised based on size groupings of < 3cm and < 2cm, breast volume ratio (as displayed in Table 4), middle range breasts, where breast volume measures between 501 – 1000ml (5/14 non‐compliant plans) and large breast size, classified as volume larger than 1000 ml (0/14 non‐compliant plans).

### Dose metrics

Resulting mean dose metrics for target structures and OARs for all patients are displayed in Table 5. Contralateral lung metrics and the volume of PTV receiving 120% of prescribed dose (PTV D_120%_) were not assessed due to prevalence of zero values. There were no statistically significant differences found between any of the dose metrics when comparing MRI to CT. Statistically significant differences were found between supine and prone positions for the volumes of contralateral breast that received 3% of the prescribed dose (p = 0.011) and volume of the ipsilateral lung that received 30% of the prescribed dose (*P* < 0.001).

**Table 5:** Dosimetric comparisons between imaging modality and position for each dose limitation and OAR

Structure	Metric	Imaging Modality (*n* = 86)					Position (*n* = 76)				
		MRI		CT			Supine		Prone		
		Mean	±SD	Mean	±SD	*P* value	Mean	±SD	Mean	±SD	P value
Contralateral Breast	D3_%(Gy)_	0.14	0.62	0.24	0.55	0.4	0.10	0.35	0.33	0.78	0.011*
	D_5%(Gy)_	0.05	0.33	0.04	0.14	0.33	0.03	0.13	0.07	0.36	0.979
Contralateral Lung	D_5%(Gy)_	0.00	0.00	0.00	0.00		0.00	0.00	0.00	0.00	
CTV	D_95%(Gy)_	97.03	4.06	96.84	4.30	0.64	97.33	4.32	96.50	4.24	0.15
Heart	D_5%(Gy)_	3.83	7.39	4.34	8.95	0.97	5.18	10.44	3.62	6.32	0.889
IpsiBreastPRV	D_50%(Gy)_	36.45	11.23	38.77	10.55	0.38	37.29	10.40	35.75	10.86	0.228
	D_95%(Gy)_	11.90	5.60	12.29	6.46	0.94	11.17	6.07	11.75	5.55	0.329
	D_100%(Gy)_	3.67	3.67	3.98	3.80	0.75	3.37	3.29	2.96	2.86	0.506
Ipsilateral Lung	D_30%(Gy)_	7.20	9.12	7.26	8.40	0.89	10.85	11.37	3.41	3.93	<0.001*
Normal Tissue	Max(Gy)	39.89	0.53	39.95	0.58	0.43	39.85	0.50	39.83	0.57	0.95
PTV EVAL	2cc(%)	103.27	1.08	103.58	1.07	0.11	103.19	1.07	103.43	1.11	0.301
	D_120%(Gy)_	0.00	0.00	0.00	0.00		0.00	0.00	0.00	0.00	
	D_90%(Gy)_	98.16	2.92	97.77	3.12	0.53	98.18	3.01	97.38	3.24	0.11

OAR: organ at risk, CTV: clinical target volume, PTV EVAL: planning target volume used for evaluation , IpsiBreastPRV: ipsilateral breast planning reference volume excluding the PTV structure, Normal Tissue: Patient excluding PTV, D_X%_: the dose received to X% of the structure, as a percentage of the prescribed dose. *statistical significance after chi‐squared testing.

## Discussion

At the time of analysis, no previous publications have, to our knowledge, combined MRI derived target volumes (vs. CT) in PBI planning. Imaging modality did not appear to significantly impact OAR doses or the rate of compliance in this cohort, and PTV EVAL sizes did not vary enough to display statistical significance across competing modalities. However, previous investigations using the same patient data sets have demonstrated that STAPLE SC volumes were smaller based on MRI delineation compared to CT.^24^ This suggests that potential nuances in size when targets are based on differing imaging modalities may be non‐consequential when PTV expansions are applied. As seroma volumes were expanded to PTVs and clipped 5 mm within the patient contour, this study found no significant difference to OAR doses if target volumes are derived from MRI or CT. Although this study has not shown any dosimetric difference in a comparison of MRI and CT derived target volumes, it has shown that the integration of MRI derived target volumes may be unlikely to affect planning dosimetry, resulting in the modality being able to be utilised for its soft tissue visualisation properties,^18^, ^24^ without impacting current clinical practice. However, the impact of modality on target expansion in PBI planning should be continued to be investigated.

In this cohort, OAR dosimetry was more favourable for the contralateral breast in the supine position and more favourable for the ipsilateral lung in the prone position. This supports the existing literature that reports that prone positioning is able to reduce overall mean lung dose^19^ and statistical significance found in other investigations where dose to the lungs is investigated based on the patients method of positioning. ^4^, ^21^ The dose to the contralateral breast may have been influenced by issues with patient positioning within the available number of retrospective data sets. Issues with contralateral breast position and reproducibility in the prone position is supported by previous investigations.^21^, ^27^ Unlike the results from our investigation, which showed no significance between dose to the heart and positioning, significant benefit has been found in reduction of heart dose in the prone position,^19^ suggesting that further investigations into the prone position and reductions to heart dose should be considered. However, there is conflicting data in regard to the best positioning method for heart sparing, as some investigations have found higher doses when using the prone position.^21^, ^27^

Within this group of patients, the PBI volume ratio data were collected for analysis, but not used as the basis of exclusion. TROG 06.02 credentialing publications have demonstrated feasibility of selecting patients for PBI based on the PBI breast volume ratio.^32^ This ratio was set at 0.25; however, other publications have not considered for PBI breast volume ratio in patient selection.^8^, ^11^, ^12^, ^14^, ^19^ Ultimately, there was no significance between ratio below 0.25 and compliance or conversely ratio larger than 0.25 and non‐compliance in this cohort. This indicates that patients should not be excluded for PBI on the basis of PBI breast volume ratio. Patients may also still be eligible for PBI if the seroma is larger than 2 cm, which has been used as selection criteria in recent clinical trials^14^, ^30^, ^32^ and < 3 cm as per international consensus statements and guidelines.^5^, ^6^, ^13^

Our study reported a 21% incidence of UIQ targets for the imaging study and 26% for the positioning component, which is higher than the 16% reported elsewhere.^33^ This increased proportion of patients with tumours in the UIQ likely influenced the incidence of heart dose and non‐compliance in this cohort, due to geographical proximity, supporting the findings from this publication that demonstrated an association between UIQ location and non‐compliance. There was also a proven association between smaller breast size (< 500 ml) and non‐compliance, which is a measure seldom reported in PBI literature, other than credentialing papers for a PBI breast volume ratio.^13^, ^14^, ^30^ Therefore, it may be beneficial to consider topography and breast size in patient selection for PBI. However, further investigation on the basis of patient selection or exclusion based on this criterion is warranted.

Due to study design, there was no minor violation metrics for dose to OARs. This was chosen to limit dose to OARs as much as possible and align with previous publications.^30^ However, the RTOG 0319^10^, ^11^ trial allowed differing dose limitations depending on tumour laterality, specifically for the heart, with a small amount of their cohort resulting in non‐compliance due to dose to the heart, 7% in minor violation and 2% in major violation.^11^ RTOG 0319^10^, ^11^ study design differs to more recent publications, such as TROG 06.02^30^, where dose constraints were not impacted by laterality of tumour. This may have impacted the cohort within our investigation. Every plan that was non‐compliant had been impacted by heart limitations and high proportion of UIQ tumours. Strict dose constraints are necessary in limiting potential radiation induced side effects, such as cardiac disease.^3^ However, using these constraints for left sided tumours resulted in high non‐compliance within this cohort, which has not been in the same magnitude in previous publications.^11^ This suggests when using stricter constraints PBI may not be feasible for all patients who otherwise meet selection criteria when employing a 3DCRT technique. Using inverse planning techniques such as intensity modulated radiation therapy (IMRT) and volume modulated radiation therapy (VMAT) could be explored as alternative methods of treatment delivery, if heart dose constraints were unable to be met using 3DCRT techniques.

This investigation was limited by the number of retrospective patient data sets available for examination (n = 35) and PBI eligibility criteria in image modality comparisons (n = 24) and in positioning (n = 19). However, the results from this small sample size may be beneficial in the ongoing investigation of MRI integration into radiotherapy practice, as some of the experiences learnt may be preserved to further equivalence studies.

Additionally, patients with a large body habitus had been removed from the initial cohort due to physical limitations of the MRI bore size. Therefore, these results may not apply to patients with a larger body habitus, for which prone positioning may be the most beneficial.^20^

To our knowledge, no previous publications have combined MRI derived target volumes (vs. CT) and differences in positioning methods (supine vs. prone) for PBI. Notably, positioning seems to influence dose to OARs. This investigation reports that contralateral breast doses are reduced in the supine position, ipsilateral lung is reduced in the prone position, and there was no significance in dose delivered to the heart in either position.

## Conclusion

In respect to our primary aims, PBI plans with PTVs generated from MRI indicated no statistical difference with respect to PTVs and OAR doses or plan compliance and OAR doses were not lowered when PBI planning was based on target volumes derived from MRI. However, statistical significance was found with different methods of positioning, where dose delivered to contralateral breast was lower in the supine positioning and ipsilateral lung doses were reduced in the prone position. Although there was no proven superiority of MRI derived target volumes, the integration of the modality remains the topic of intense interest with recent investigations into breast radiotherapy planning, single dose PBI treatment and the ongoing development of breast MRI imaging sequences. ^17^, ^18^, ^22^, ^23^ However, more research in a clinical setting is needed to validate the modalities integration.

## Conflict of Interest

The authors declare no conflict of interest.
